# A Pancreatic Ductal Adenocarcinoma Diagnostic System Using Serum Extracellular Vesicle Detection with Optimized Lectin Combination Using Machine Learning

**DOI:** 10.3390/cancers18060924

**Published:** 2026-03-12

**Authors:** Tatsuya Kawakami, Sho Uemura, Masayuki Ono, Katsue Horikoshi, Atsushi Kuno, Ayumi Kashiro, Kazufumi Honda, Kengo Nagashima, Kazuki Kumada, Masaya Munekage, Satoru Seo, Kaoru Furihata, Mutsuo Furihata, Koichi Honke, Minoru Kitago, Yuko Kitagawa, Makoto Suematsu, Makoto Itonaga, Yasuaki Kabe

**Affiliations:** 1 Future Creation Research Laboratory, JVCKENWOOD Corporation, Yokohama 221-0022, Japan; kawakami.tatsuya@jvckenwood.com (T.K.); ono.masayuki@jvckenwood.com (M.O.); horikoshi-katsue@jvckenwood.com (K.H.); 2 Department of Surgery, Keio University School of Medicine, Tokyo 160-8582, Japan; shouemura0419@keio.jp (S.U.); dragonpegasus@keio.jp (M.K.); kitagawa.a3@keio.jp (Y.K.); 3 Cellular and Molecular Biotechnology Research Institute, National Institute of Advanced Industrial Science and Technology (AIST), Tsukuba 305-8560, Japan; atsu-kuno@aist.go.jp; 4 Department of Bioregulation, Graduate School of Medicine, Nippon Medical School, Tokyo 113-8602, Japan; a-kashiro@nms.ac.jp (A.K.); k-honda@nms.ac.jp (K.H.); 5 Biostatistics Unit, Clinical and Translational Research Center, Keio University, Tokyo 160-8582, Japan; nshi@keio.jp; 6 Tohoku Medical Megabank Organization, Tohoku University, Sendai 980-8577, Japan; kazuki.kumada.c7@tohoku.ac.jp; 7 Department of Surgery, Kochi Medical School, Kochi University, Nankoku 783-8505, Japan; m-munekage@kochi-u.ac.jp (M.M.); rutosa@kochi-u.ac.jp (S.S.); 8 Department of Pathology, Kochi Medical School, Kochi University, Nankoku 783-8505, Japan; jm-k.furihata@kochi-u.ac.jp (K.F.); furiham@kochi-u.ac.jp (M.F.); 9 Department of Biochemistry, Kochi Medical School, Kochi University, Nankoku 783-8505, Japan; khonke@kochi-u.ac.jp; 10 Central Institute for Experimental Medicine and Life Science, Kawasaki 210-0821, Japan; gasbiology@keio.jp; 11 Healthcare Business Division, JVCKENWOOD Corporation, Yokohama 221-0022, Japan

**Keywords:** pancreatic ductal adenocarcinoma, biomarkers, glycan, lectin, extracellular vesicles

## Abstract

**Background**: Pancreatic ductal adenocarcinoma (PDAC) has one of the poorest prognoses among malignant tumors, mainly due to the difficulty of early diagnosis. Therefore, it is crucial to identify reliable blood markers for a highly sensitive diagnostic system. We previously developed a highly sensitive extracellular vesicle (EV)-counting system, which can quantify the absolute number of specific EVs in serum. In this study, a multiplex assay using lectins that recognize specific glycans on EVs in the serum of PDAC patients was performed to select the optimal lectin combination. **Methods**: The glycan alteration signature of serum EVs from patients with PDAC was analyzed using a lectin-based multiplex assay combined with the EV-counting system. The optimal lectin combination that recognizes PDAC-specific changes was selected using machine learning analyses (support vector machine) for high diagnostic performance across independent patient cohorts. **Results**: An optimal lectin combination, Jacalin and Agaricus bisporus agglutinin (ABA), for PDAC detection was identified using machine learning analysis. This lectin-based system, reflecting changes in Jacalin/ABA binding, showed significantly higher diagnostic performance (area under the curve [AUC] = 0.890 and 0.971) than that of the conventional diagnostic marker carbohydrate antigen 19-9 (CA19.9; AUC = 0.752). Notably, the system achieved an AUC of 0.870 in patients with the stage I disease. **Conclusions**: These findings highlight the potential of a serum EV-based diagnostic system leveraging Jacalin and ABA glycan recognition for the early detection of PDAC.

## Simple Summary

This study aimed to establish a novel diagnostic system for pancreatic ductal adenocarcinoma (PDAC) by identifying extracellular vesicles (EVs) with specific glycan markers in the blood using a highly sensitive EV-counting system that we previously developed. We performed a multiplex assay using lectins that recognize specific glycans on EVs in the serum. The glycan alteration signature of serum EVs from PDAC patients was analyzed using machine learning (support vector machine), resulting in the identification of an optimal lectin combination, Jacalin and Agaricus bisporus agglutinin (ABA), that achieved high diagnostic performance of PDAC. This lectin-based system, reflecting changes in Jacalin/ABA binding, demonstrated significantly higher diagnostic performance (area under the curve [AUC] = 0.890 and 0.971). Notably, the system achieved an AUC of 0.870 in patients with the stage I disease. These findings highlight the potential of a serum EV-based diagnostic system leveraging Jacalin and ABA glycan recognition for the early detection of PDAC.

## 1. Introduction

Among common malignancies, pancreatic ductal adenocarcinoma (PDAC) has one of the poorest prognoses [1]. In 2024, PDAC was ranked as the fourth leading cause of cancer-related deaths in the United States, with a 5-year survival rate of only 13%, largely owing to its highly aggressive nature and the difficulty of early diagnosis [2]. Surgical resection remains the only curative treatment for patients with PDAC without distant metastasis; however, only about 20–30% of patients are eligible for resection at the time of diagnosis [3]. Consequently, there is a need to develop reliable screening strategies for PDAC to improve survival outcomes.

Carbohydrate antigen 19-9 (CA19-9) is widely used as a serological marker for PDAC diagnosis [4]; however, its diagnostic reliability is limited. While several methods for detecting PDAC-derived molecules in body fluids have been reported [5,6], no sufficiently reliable method for PDAC diagnosis has been established. Therefore, identifying a reliable screening marker remains crucial for improving PDAC diagnosis.

Tumor cells often have glycans with diverse structures, including increased sialylation and/or fucosylation, truncated or branched O-glycans, and altered mucin glycosylation [7]. Glycosphingolipids such as gangliosides, di-sialogangliosides, and tri-sialogangliosides have also been associated with malignancy [7]. Notably, multiplexed lectin assays have been reported to improve diagnostic performance in hepatocellular carcinoma [8], and glycan alterations have also been observed in PDAC [9]. Indeed, altered glycosylation forms the basis of several blood markers, including CA19-9 [4], carcinoembryonic antigen (CEA) [10,11], and Duke pancreatic antigen 2 [12]. However, the sensitivity and specificity of these biomarkers remain inadequate for PDAC screening.

Extracellular vesicles (EVs) are cell-secreted membranous vesicles with diameters of approximately 50–150 nm that contain genetic biomaterials such as nucleic acids and proteins and contribute to intercellular communications [13,14]. EVs are involved in the regulation of numerous physiological processes, including cancer progression and metastasis [15,16], and several cancer-related EVs containing specific marker antigens have been reported [17,18,19]. In PDAC sera, several specific EVs containing proteins such as ephrin receptor A2, epidermal growth factor receptor, glypican-1, and epithelial cell adhesion molecule have been reported to be elevated; however, their sensitivity and specificity are insufficient for use in PDAC screening [20].

We previously developed an EV-counting system, ExoCounter, that combines nanobead properties with optical disk technology to quantify the absolute number of specific EVs in serum [21]. Using a lectin microarray, we recently demonstrated that multiple lectin-specific EV populations are altered in patients with PDAC, and that EVs positive for the O-glycan-binding lectins Agaricus bisporus agglutinin (ABA) and Amaranthus caudatus agglutinin (ACA) are elevated in PDAC serum when measured using ExoCounter [22]. However, because the diagnostic sensitivity of ABA or ACA alone for PDAC is insufficient, further comprehensive analyses using multiple lectins are needed to develop a more reliable diagnostic system.

In this study, we analyzed the glycan alteration signature of serum EVs from patients with PDAC using a lectin-based multiplex assay combined with the ExoCounter platform. Machine learning analysis of PDAC-specific alterations revealed that the combination of Jacalin and ABA lectins has high diagnostic performance across independent patient cohorts.

## 2. Materials and Methods

### 2.1. Antibodies and Lectins

Anti-CD9 (clone HI9a) and isotype control IgG1 (clone MG1−45) antibodies were purchased from BioLegend Inc. (San Diego, CA, USA) for conjugation with nanobeads. Anti-CD9 antibodies (clone 12A12) were purchased from CosmoBio Co. (Tokyo, Japan) for disk coating. The following lectins were used: ABA (J-Chemical, Inc., Tokyo, Japan); Datura stramonium agglutinin (DSA), Urtica dioica agglutinin (UDA), and V (ACA) (EY Laboratories, San Mateo, CA, USA); Aleuria Aurantia Lectin (AAL), Lycopersicon esculentum lectin (LEL), Lotus tetragon olobus lectin (LTL), and Solanum tuberosum lectin (STL) (Vector Laboratories, Newark, CA, USA); Agrocybe cylindracea galactose-binding lectin (ACG), Sambucus sieboldiana agglutinin (SSA), Lens culinaris agglutinin (LCA), Peanut agglutinin (PNA), and Lathyrus sativus lectin-N (LSL-N) (Fujifilm Wako Pure Chemical Corp., Osaka, Japan); Jacalin (Geno Technology, Inc., St. Louis, MO, USA); Concanavalin A (ConA) (LKT Laboratories, Inc., St. Paul, MN, USA).

### 2.2. Cell Culture

BxPC-3, CFPAC and Capan-1 cells were purchased from ATCC. BxPC-3 cells were maintained in Roswell Park Memorial Institute 1640 medium supplemented with 10% fetal bovine serum (FBS) at 37 °C in a 5% carbon dioxide (CO_2_)-humidified incubator. CFPAC and Capan-1 cells were maintained in Improved Minimum Essential Medium containing 10% (for CFPAC) or 20% (for Capan-1) FBS at 37 °C in a 5% CO_2_ humidified incubator. When cells reached semi-confluence, they were washed twice with phosphate-buffered solution (PBS) and cultured in serum-free medium for 48 h. Culture media were subsequently collected and centrifuged at 13,000× *g* for 30 min prior to analysis with the ExoCounter (JVCKENWOOD, Yokohama, Japan).

### 2.3. Clinical Samples

Cohort 1: Sera from 42 patients with PDAC obtained from Keio University Hospital. Characteristics of patients were summarized in Table S1.

Cohort 2: Sera from 20 patients with PDAC and 20 normal control (NC) collected from prospective cohorts at the National Cancer Center Research Institute and National Center Biobank Network.

Other cancers: Sera from patients with lung cancer (LC), colorectal cancer (CC), gastric cancer (GC), and breast cancer (BC) were obtained from Bio Bank Japan (n = 10 for each cancer type).

Normal Control (NC): NC samples (n = 41)were obtained from the Tohoku Medical MegaBank Organization, Sendai, Japan.

All blood samples were stored at −80 °C until use.

### 2.4. Preparation of Antibody-Conjugated Nanobeads

Anti-CD9 antibody-conjugated ferrite-glycidyl methacrylate (FG) beads were prepared based on the method described elsewhere [21]. Carboxylated FG beads (1 mg, approximately 2 × 1011 particles) and antibodies (50 μg) were mixed in 50 μL of 50 mM acetate buffer (pH 4.5) containing 30 mM 1-ethyl-3-(3-dimethylaminopropyl)carbodiimide and subsequently incubated at 37 °C for 1 h. After removing the unbound antibodies, amino polyethylene glycol was added, and the cells were further incubated for 3 h at room temperature. Subsequently, the buffer was replaced with 500 μL of 10 mM N-2-hydroxyethylpiperazine-N’-2-ethanesulfonic acid (pH 7.9) containing 1 mM ethylenediaminetetraacetic acid and 0.1% Tween-20 and stored at 4 °C until use.

### 2.5. Isolation of EVs from Serum

EVs were isolated via SEC using a single 70 nm qEV column (IZON, Christchurch, New Zealand). The qEV column was equilibrated with 4 mL PBS. Thereafter, serum samples (100 μL) were centrifuged at 2000× *g* at 4 °C for 5 min, and the supernatant was applied to the column. The first 1.1 mL of PBS was discarded, and the EV fraction was eluted with 600 μL of PBS.

### 2.6. Validation of the Isolated EVs

The size distribution of isolated EVs was analyzed using a NanoSight LM10 (Malvern Panalytical, Malvern, UK). EVs were diluted 10-fold and injected into the sample chamber. Measurements were performed at room temperature for 60 s, and data were captured and analyzed using NTA 3.2 Dev Build 3.2.16 (Malvern Panalytical). The isolated EV morphologies were examined using STEM (S-5200, Hitachi High-Technologies Co., Tokyo, Japan). EVs in PBS were mixed 1:1 with 4% paraformaldehyde and deposited onto Parafilm. Transmission electron microscopy grids coated with a colloidal film were placed on the drops for 30 min, then stained with 3% phosphotungstic acid for 30 s. Grids were washed with deionized water, and images were acquired using STEM at an acceleration voltage of 30 kV.

### 2.7. Quantification of EVs Using ExoCounter

Serum EV fractions were analyzed with an ExoCounter using the following protocol: An optical disk was attached to a removal plate containing 16 wells for sample injection. Each well was coated with 5 μg/mL of lectin or antibody (anti-CD9 antibody) in carbonate buffer (pH 9.6) for 30 min at 37 °C. After washing with PBS containing 0.05% Tween 20 (PBS-T), the wells were blocked with PBS-T containing 1% Carbo-free blocking solution (Vector Laboratories, Inc., USA) (for lectins) or 0.1% skim milk (for anti-CD9 antibody) for 30 min at 37 °C.

EV fractions (50 µL sample) were incubated in the wells for 2 h at 37 °C, then washed three times with PBS-T. Approximately 1.6 × 108 anti-CD9 antibody-conjugated beads in blocking solution (0.1% casein in PBS-T for lectins or 0.1% skim milk in PBS-T for anti-CD9 antibody) were added and incubated for 2 min under a magnetic field. Each well was washed sequentially with PBS-T and deionized water, dried in a thermostatic oven at 37 °C for 15 min, and lectin-positive or CD9-positive EVs were quantified using the ExoCounter.

### 2.8. ELISA (CA19-9)

Serum CA19-9 levels were measured using the TM-CA19-9 enzyme-linked immunosorbent assay kit (DRG Instruments GmbH, Marburg, Germany). Assays were performed using serum samples from Cohort 1 (NC = 34, PDAC = 31) according to the manufacturer’s instructions.

### 2.9. Statistical Analysis

Statistical analyses were performed using Bell Curve for Excel. Comparisons among PDAC stages were conducted using one-way analysis of variance, followed by multiple comparison tests with the Tukey–Kramer method.

### 2.10. Data Analysis by Machine Learning

A machine learning model built with a support vector machine (SVM), implemented in Python version 3.11 via Sci-kit learn, was used to choose the optimal combination of lectins for distinguishing pancreatic cancer patients from normal controls. The input data to the model were the normalized counts, which were the counts for each lectin divided by the number of CD9 positive exosomes, and target values (ground truth) 1 or 0 for PC or NC, respectively.

## 3. Results

### 3.1. Experimental Scheme of This Study

**Figure 1:**
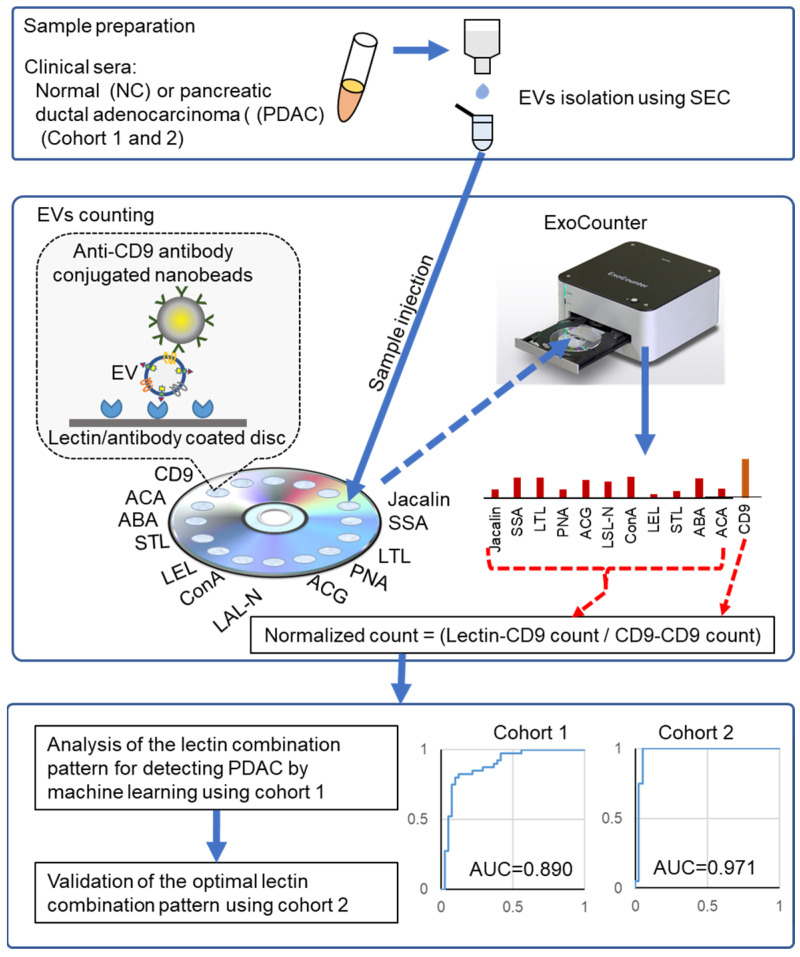
Development of a PDAC diagnostic system using EVs recognized by an optimized lectin combination. Serum from normal controls and patients with PDAC from two independent cohorts was analyzed. Serum EVs were isolated using size-exclusion chromatography. Specific EVs were captured on lectin-coated disks (Jacalin, SSA, LTL, PNA, ACG, LSL-N, ConA, LEL, STL, ABA, or ACA), labeled with anti-CD9 antibody–conjugated beads, and quantified using an ExoCounter. Counts of each lectin-positive EV were normalized to the total CD9-positive EVs. The optimal lectin combination for PDAC diagnosis was determined using machine learning based on ROC analysis (AUC) using Cohort 1 data and subsequently validated using Cohort 2 data.

Figure 1 illustrates the experimental scheme used in this study. Two independent cohorts, comprising sera from patients with PDAC and normal controls (NCs), were analyzed. Cohort 1 included 42 PDAC serum samples (Table S1) obtained from Keio University Hospital and 41 NC serum samples obtained from the Tohoku Medical MegaBank Organization, Japan. Cohort 2 included 20 PDAC serum samples obtained from prospective cohorts at the National Cancer Center Research Institute and the National Center Biobank Network.

EV fractions were isolated from serum using size-exclusion chromatography (SEC). The EV size distribution and morphology were validated using nanoparticle tracking analysis and scanning transmission electron microscopy (STEM; Figure S1A,B). Purified EVs were quantified using an exosome-counting system (ExoCounter).

Glycan-specific EVs were captured by coating 11 lectins onto the surface of an optical disk and detected by labeling with anti-CD9 antibody-conjugated nanobeads. Lectin-positive EV counts were normalized to the total EV counts captured on the optical disks coated with anti-CD9 antibody. The dataset of each lectin-positive EV from Cohort 1 was analyzed using a support vector machine (SVM)-based machine learning model. The optimal lectin combination for PDAC diagnosis was subsequently validated using Cohort 2 samples. The diagnostic performance of lectin combinations was assessed by calculating the area under the receiver operating characteristic (ROC) curve (AUC).

### 3.2. EV Detection Using Multiple Lectins

For PDAC EVs, we initially evaluated 14 lectins Sambucus sieboldiana agglutinin (SSA), Agrocybe cylindracea galactose-binding lectin (ACG), Jacalin, ACA, Peanut agglutinin (PNA), ABA, Concanavalin A (ConA), Lotus tetragon olobus lectin (LTL), Lycopersicon esculentum lectin (LEL), Solanum tuberosum lectin (STL), Aleuria Aurantia Lectin (AAL), Urtica dioica agglutinin (UDA), Lens culinaris agglutinin (LCA), and Lathyrus sativus lectin-N (LSL-N), selected based on our previous microarray analyses [22] and reports from other groups [23,24,25,26,27,28,29]. First, we evaluated nonspecific binding of anti-CD9 antibodies to lectins. LCA, AAL, and UDA showed high background counts in blank samples (Figure S1C) and were therefore excluded from subsequent measurements.

Next, using EV-enriched fractions obtained from PDAC serum, we evaluated whether EVs captured by the remaining 11 lectins could be specifically detected using anti-CD9 antibody-conjugated nanobeads. For all lectins, CD9-positive EV counts were higher than those obtained using IgG-conjugated control beads (Figure S1D). These 11 lectins were further evaluated using culture media from PDAC cell lines (Capan-1, BxPC3, and CFPAC) (Figure S1E). There was some overall variation; however, lectin-positive EV counts derived from PDAC cells were observed among the cell lines. Thus, 11 lectins (SSA, ACG, Jacalin, ACA, PNA, ABA, ConA, LTL, LEL, STL, and LSL-N) were selected for subsequent analyses (Table S2).

### 3.3. Optimization of Lectin Combination for PDAC Diagnosis Using Machine Learning

**Figure 2:**
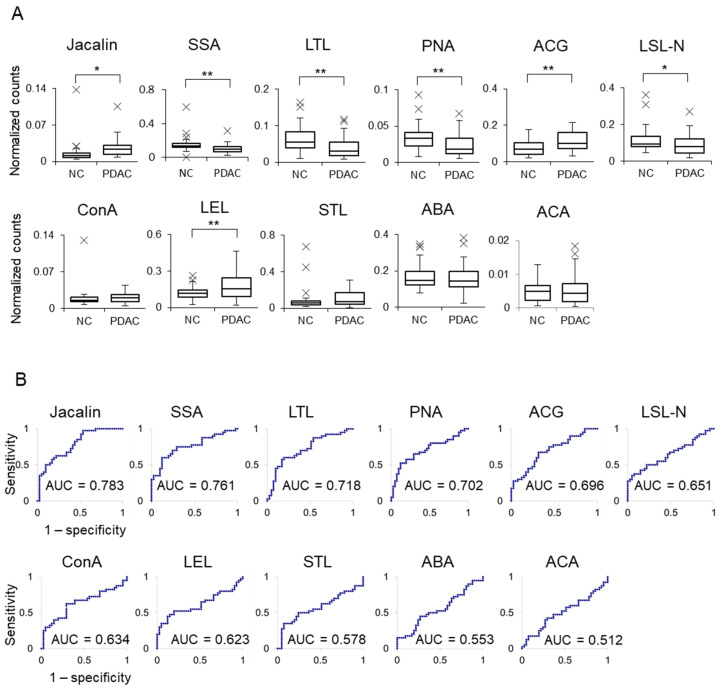
Comparison of lectin-positive EVs between NC and PDAC sera. (**A**) The specific EVs from Cohort 1 (NC, n = 41; PC, n = 40) were quantified using each lectin (Jacalin, SSA, LTL, PNA, ACG, LSL-N, ConA, LEL, STL, ABA, and ACA) with ExoCounter. Lectin-positive EV counts were normalized to CD9-positive EV counts. *p*-values were calculated using a *t*-test (* *p* < 0.05 and ** *p* < 0.01). Outliers are indicated by ×. (**B**) ROC curves for each lectin-positive EV from PDAC compared with those from NC.

First, lectin-positive EVs were measured in sera from NC (n = 41) and patients with PDAC (n = 40) in Cohort 1 using ExoCounter. Figure 2 shows the normalized counts of lectin-positive EVs (Figure 2A) and the corresponding ROC analysis-derived AUC values (Figure 2B). Among the individual lectins, Jacalin exhibited the highest AUC; however, this value was below 0.8, indicating that sufficient diagnostic performance could not be achieved using a single lectin. Therefore, we attempted to optimize diagnostic performance by analyzing combinations of multiple lectins.

**Figure 3:**
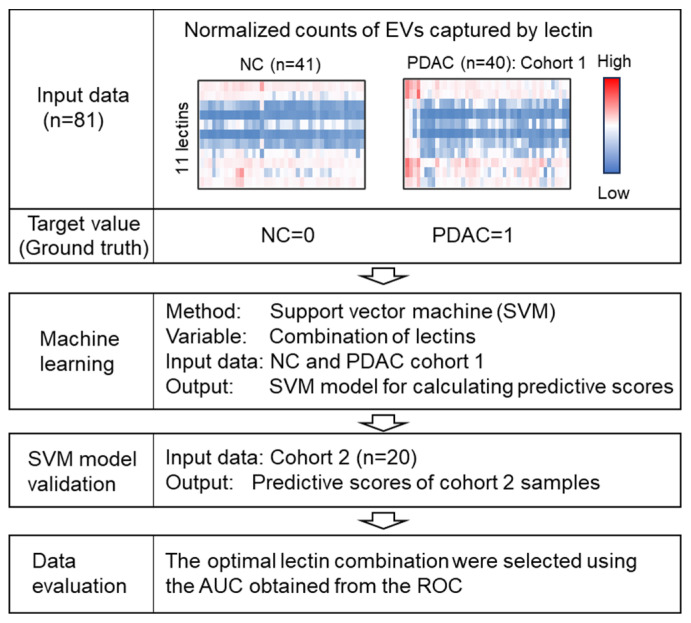
Workflow for lectin combination optimization using machine learning. To determine the optimal lectin combination for pancreatic ductal adenocarcinoma (PDAC) diagnosis, normalized counts of lectin-positive EVs from Cohort 1 were evaluated using machine learning based on a support vector machine (SVM) to generate predictive scores. The resulting model was validated using Cohort 2 data. Receiver operating characteristic (ROC) curves were calculated from the predictive scores to assess diagnostic performance.

To identify optimal lectin combinations for PDAC diagnosis, datasets of lectin-positive EV counts in serum were analyzed using an SVM-based machine learning model. The analysis workflow is illustrated in Figure 3. SVM models were trained to generate predictive scores from normalized lectin-positive EV counts, using ground-truth labels (NC = 0; PDAC Cohort 1 = 1). The resulting predictive scores were subsequently vali-dated using Cohort 2 samples (raw data are shown in Figure S2). Optimal lectin combinations were selected based on the AUC values obtained from ROC analyses. 

Table 1 shows the AUC results of single lectins (left panel) and two-lectin combinations (right panel) obtained from Cohorts 1 and 2 or a combination of both cohorts (Cohort 1 + 2). The two-lectin combinations shown represent the top 10 combinations based on AUC values in Cohort 1 + 2. Notably, most high-performing combinations included Jacalin.

**Figure 4:**
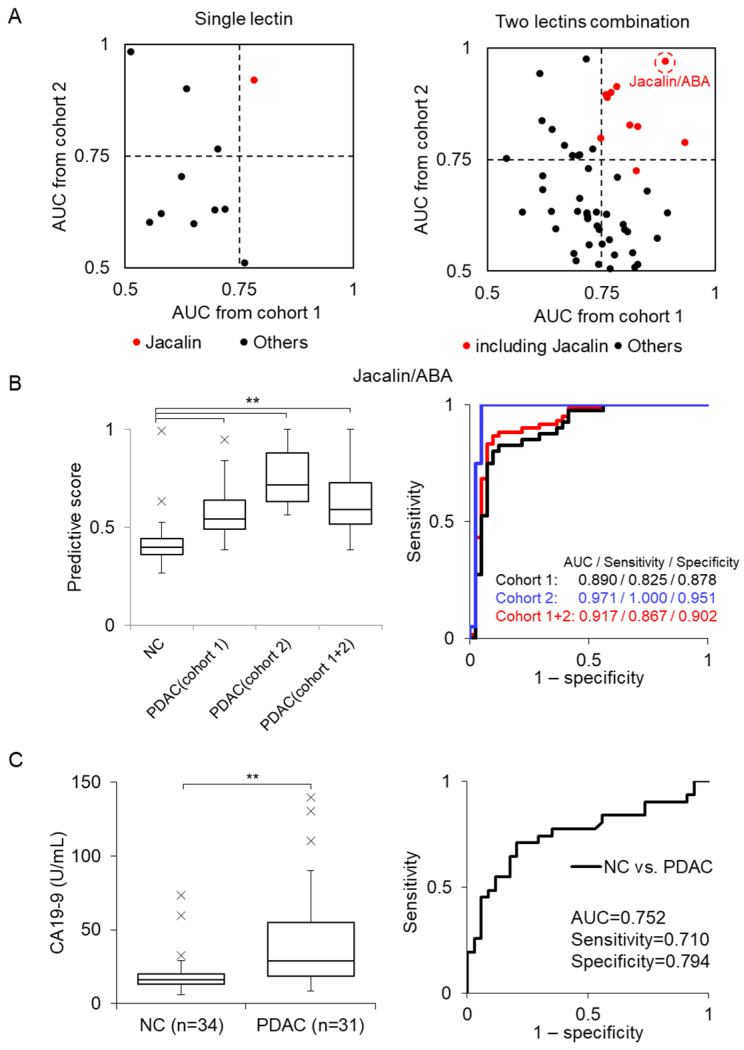
Diagnostic performances for PDAC using the Jacalin/ABA combination. (**A**) Scatter plots showing the correlation between AUCs from Cohorts 1 and 2 for single lectins (left panel) and two-lectin combinations (right panel). Red dots represent Jacalin or a combination containing Jacalin. (**B**) Box plots (left panel) and ROC curve (right panel) for sera from Cohort 1 (n = 40), Cohort 2 (n = 20), and the combined cohort (Cohort 1 + 2; n = 60) against NC (n = 41) using Jacalin/ABA. AUC, sensitivity, and specificity are indicated. (**C**) Box plots (left panel) and ROC curve (right panel) for CA19-9 levels measured using ELISA in Cohort 1 (NC = 34, PDAC = 31). (**B**,**C**) Outliers are indicated by ×. *p*-values were calculated using a *t*-test (** *p* < 0.01).

Figure 4A shows the comparison of AUC values between Cohorts 1 and 2 in single lectins and two-lectin combinations, indicating that combinations containing Jacalin (red dot) achieved higher AUCs. Among the two-lectin combinations, the Jacalin/ABA combination yielded AUC values higher than 0.89 across Cohorts 1 and 2, as well as the combined cohort (Cohort 1 + 2), outperforming the single lectin Jacalin (Table 1). Furthermore, analysis of three- and four-lectin combinations showed that combinations containing Jacalin/ABA achieved similarly high AUCs (Figure S3). Because these results were comparable to those of the two-lectin Jacalin/ABA combination, subsequent analyses were performed using this combination. AUC with Jacalin/ABA combination were 0.890 (95% CI: 0.816–0.965), 0.971 (95% CI: 0.924–1.018), and 0.917 (95% CI: 0.855–0.979) for Cohorts 1, 2 and 1 + 2, respectively.

**Figure 5:**
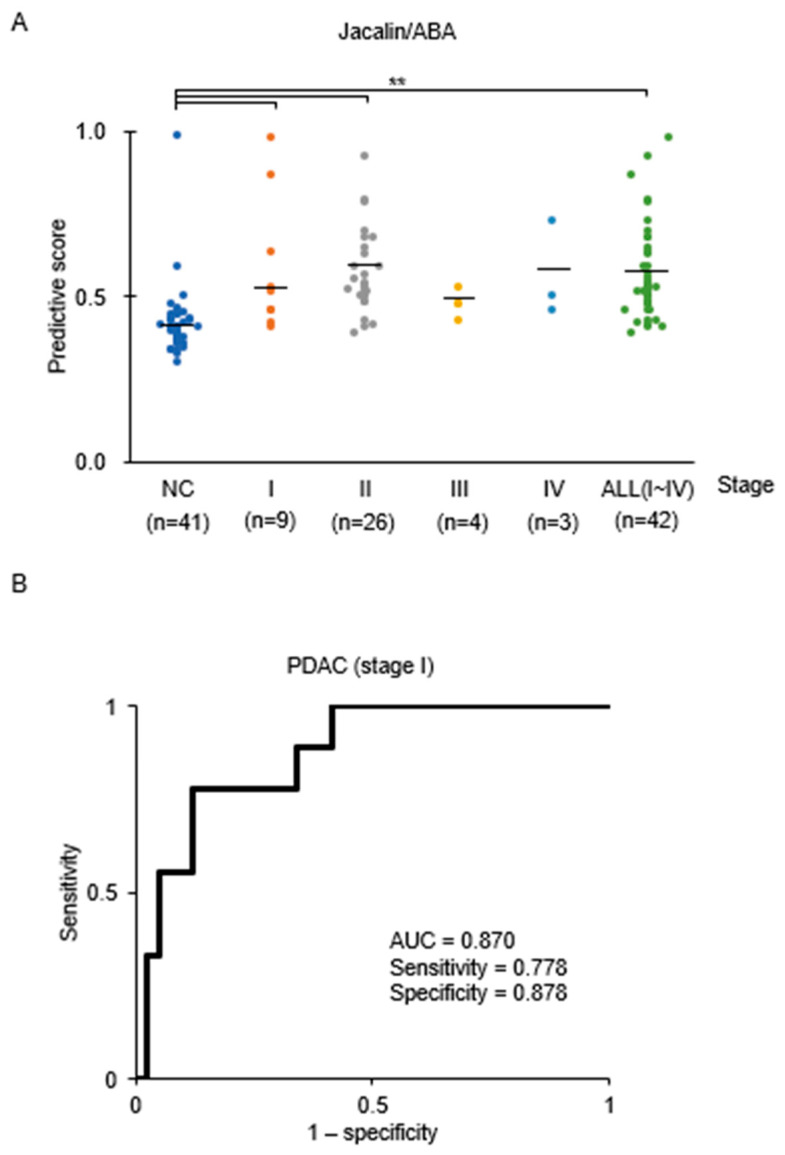
Detection of PDAC at different stages using Jacalin/ABA and comparison of Jacalin/ABA-based detection across multiple cancer types. (**A**) Dot plots of predictive scores for each PDAC stage. Sample numbers: NC, 41; Stage I, 9; Stage II, 26; Stage III, 4; and Stage IV, 3. Bars indicate the mean. Statistical analysis was performed using ANOVA with the Tukey–Kramer test (** *p* < 0.01). (**B**) ROC curve showing the comparison between Stage I PDAC and NC. AUC, sensitivity, and specificity at 0.870, 0.778, and 0.888, respectively.

Figure 4B shows the box plots of the SVM predictive scores and the corresponding ROC curves obtained using the Jacalin/ABA combination. For comparison, we also measured serum CA19-9 levels in a subset of Cohort 1 samples (NC = 34; PDAC = 31) (Figure 4C). The AUC of the CA19-9 test was 0.752, which was comparable to the previously reported values (0.76–0.78) [30]. The difference between the AUC of Jacalin/ABA model and CA19-9 test was assessed using DeLong’s test (*p*-value of 0.028). Precision–Recall AUC (PR-AUC), calibration curves, and decision-curve analysis were also performed for Jacalin/ABA combination and CA19.9 (Figure S4). The Jacalin/ABA-based EV detection system showed significantly higher performance than that of the CA19-9-based detection system. We further analyzed the performance of the Jacalin/ABA combination across different PDAC stages in Cohort 1. Significant differences were observed between NC and PDAC groups at stages I and II (Figure 5A). ROC analysis between NC and stage I PDAC yielded an AUC value of 0.870 (95% CI: 0.755–0.979) (Figure 5B), indicating its potential for early-stage PDAC diagnosis.

### 3.4. Detection of Other Cancers Using the Jacalin/ABA Combination

**Figure 6:**
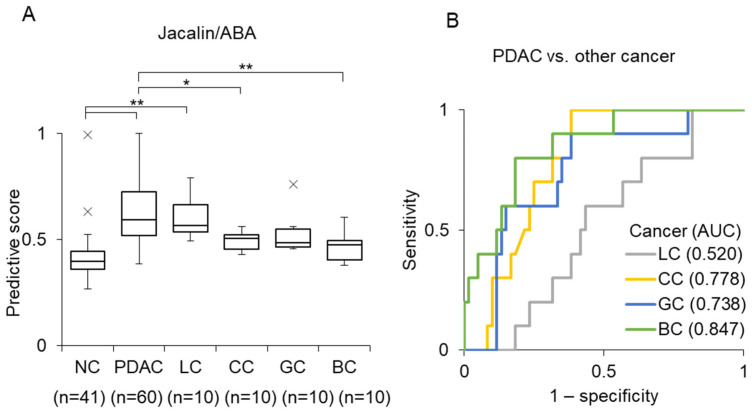
Comparison of Jacalin/ABA-based detection across multiple cancer types. (**A**) Box plots for predictive scores for EVs isolated from sera of NC (n = 41) and patients with PDAC (n = 60), lung cancer (LC) (n = 10), colorectal cancer (CC) (n = 10), gastric cancer (GC) (n = 10), and breast cancer (BC) (n = 10) using Jacalin/ABA. Outliers are indicated by ×. Statistical analysis was performed using ANOVA with the Tukey–Kramer test (* *p* < 0.05, ** *p* < 0.01). (**B**) Comparison of AUCs between PDAC and other cancers.

We next examined the performance of the Jacalin/ABA combination using sera from patients with PDAC, lung cancer (LC), colorectal cancer (CC), gastric cancer (GC), and breast cancer (BC) (Figure S5). The predictive scores for these cancer types are shown in Figure 6A. PDAC samples exhibited significantly higher predictive scores than CC or BC samples did. Furthermore, ROC analysis revealed that the AUC values for distinguishing PDAC from other cancers were 0.847 for BC, 0.777 for CC, and 0.738 for GC, whereas the AUC for LC was markedly lower (0.552), indicating limited discrimination between PDAC and LC using this model (Figure 6B).

To evaluate correlations among lectin-positive EVs across cancer types, we generated a heat map and performed hierarchical clustering using EV data measured with 11 lectins from PDAC, LC, CC, GC, BC, and NC sera (Figure S6A). Principal component analysis of lectin-specific EV profiles further indicated that Jacalin and ABA contributed independently to sample discrimination (Figure S6B).

## 4. Discussion

Cancer cell-derived, EV-specific biomarkers circulating into the bloodstream represent promising targets for liquid biopsy-based diagnostics. Therefore, accurate and quantitative detection of tumor-specific EVs is essential for reliable cancer diagnosis [18,31]. Previously, we reported that ABA- and ACA lectin-positive EVs were elevated in the serum of patients with PDAC using the ExoCounter platform [22]. Our lectin microarray analysis further showed that various lectin-specific EVs were altered in patients with PDAC [22,32]. In this study, we performed a comprehensive quantitative analysis of lectin-positive EVs using ExoCounter to improve PDAC diagnosis by identifying optimal lectin combinations. By systematically evaluating all possible lectin combinations using machine learning, we identified Jacalin/ABA as the optimal combination across two independent cohorts. This combination significantly outperformed the conventional biomarker CA19-9 and enabled highly sensitive PDAC diagnosis, including early-stage disease.

Alterations in glycosylation, both quantitative and qualitative, are well known to be associated with carcinogenesis and tumor progression [33,34]. In PDAC, reported altered glycosylations include truncated O-glycans, increased sialylation, and branched and/or fucosylated N-glycans [35,36]. Lectins are therefore widely used to detect altered glycosylation patterns in PDAC [37,38]. In this study, we showed that the Jacalin/ABA combination provides robust diagnostic performance across independent cohorts in PDAC. Consistent with our findings, we previously showed that ABA-positive EVs were elevated in the serum of patients with PDAC and that PDAC lesions are specifically stained with ABA [22]. Furthermore, in our prior lectin array screening, we identified Jacalin-positive EVs as a candidate PDAC-specific serum marker [22]. These results are consistent with findings from Baldus et al., who reported that PDAC lesions were specifically stained with Jacalin [39].

Jacalin and ABA are well known for their ability to recognize O-glycans with diverse glycan motifs [24] (Table 1). Jacalin preferentially binds to truncated O-glycans, including core 1 and 3 structures [40], whereas ABA recognizes elongated O-glycans as well as terminal GlcNAcβ residues of N-glycans [24]. Therefore, the complementary recognition of different glycosylation types likely underlies the high diagnostic performance achieved by their combination. Excessive or aberrant O-glycosylation has been implicated in multiple stages of carcinogenesis and tumor progression [33,34]. In PDAC cells, abnormal O-glycosylation of mucins such as MUC1 has been observed during tumor formation and progression, including in inflammation-associated precancerous lesions [41]. The specific antigens that bind to Jacalin/ABA-positive glycans remain unclear; however, mucin proteins such as MUC1 are plausible candidates.

We also evaluated the detection performance of Jacalin/ABA across multiple cancer types (Figure 6). Compared with that for PDAC, the diagnostic performance was very low for LC (AUC = 0.552), indicating limited discrimination between PDAC and LC with this model. While the underlying reason remains unclear, Jacalin/ABA-positive EVs were significantly increased in LC compared with those in NC, suggesting that LC may release them in a manner similar to that in PDAC. Further optimization using machine learning may enable improved discrimination among different cancer types.

This study has some limitations. First, all analyzed samples were obtained from patients with established PDAC diagnoses, limiting assessment of true screening performance in asymptomatic populations. Second, although two independent cohorts were analyzed, all participants were Japanese, which may introduce selection bias, and clinicopathological backgrounds were not matched between the two cohorts. Third, we used same NC samples for the SVM model training and validation, because it was difficult to collect another NC samples comparable to the PDAC samples for both cohorts. This may affect the results. Fourth, the specific glycan structures and glycosylated antigens in PDAC-derived EVs remain to be elucidated. Fifth, the CA19-9 measurement was performed using the serum samples only in Cohort 1. Sixth, in this study, we obtained significant results from a diagnostic performance by detecting the CD9-positive EVs. Detection of other EV markers such as CD63 and CD81 besides CD9 may verify the diagnostic performance. Seventh, further evaluation of sample preparation including size exclusion chromatography is required by detecting EV markers such as CD9, CD63, or CD81, as well as negative controls such as albumin and apoproteins. Eighth, to establish a reliable biomarker, further validation is required. This includes evaluation of analytical performance such as detection specificity (e.g., glycan specificity, etc.), reproducibility, dilution linearity, spike-in recovery, and freeze–thaw stability. Nineth, further evaluation is required to verify the specificity in a wide range of other cancer types, as well as in benign pancreatic diseases such as chronic pancreatitis, intraductal papillary mucinous neoplasm (IPMN), etc. Tenth, assessment of potential effects of medications is necessary in future studies. Finally, further analysis is required to determine the optimal lectin combination for detecting other cancer types including lung cancer.

## 5. Conclusions

This study shows highly sensitive detection of PDAC-specific EVs in sera using the optimal lectin combination of Jacalin and ABA. While further validation is needed, this lectin-based EV-counting system shows considerable potential as a diagnostic screening tool for early PDAC detection.

## Figures and Tables

**Table 1:** AUC values for single lectins and two-lectin combinations. AUC values for individual lectins were calculated from the normalized EV counts in Cohort 1, Cohort 2, and the combined dataset (Cohorts 1 + 2) (left panel). AUC values for two-lectin combinations were calculated using an SVM model trained using NC and Cohort 1 (right panel). The table represents the highest-performing results (top 10 for two-lectin combinations) based on AUC values from the combined dataset (Cohorts 1 + 2). * 95% confidence intervals with Jacalin/ABA were 0.890 (95% CI: 0.816–0.965), 0.971 (95% CI: 0.924–1.018) and 0.917 (95% CI: 0.855–0.979) for cohorts 1, 2 and 1 + 2, respectively.

Lectin	Cohort 1	Cohort 2	Cohort 1 + 2	Lectin		Cohort 1	Cohort 2	Cohort 1 + 2
Jacalin	0.783	0.920	0.828	Jacalin	ABA	0.890 *	0.971 *	0.917 *
ConA	0.634	0.900	0.722	Jacalin	SSA	0.933	0.788	0.885
SSA	0.761	0.511	0.678	Jacalin	LTL	0.830	0.824	0.828
ACA	0.512	0.984	0.654	Jacalin	ACA	0.784	0.913	0.827
LEL	0.623	0.704	0.650	Jacalin	LSL-N	0.812	0.828	0.817
LTL	0.718	0.631	0.602	Jacalin	STL	0.771	0.900	0.814
STL	0.578	0.621	0.592	Jacalin	LEL	0.760	0.896	0.806
ACG	0.696	0.629	0.588	Jacalin	ConA	0.762	0.889	0.804
ABA	0.553	0.601	0.569	ConA	ABA	0.717	0.976	0.803
LSL-N	0.651	0.599	0.568	ConA	SSA	0.850	0.679	0.793
PNA	0.702	0.765	0.546					

## Data Availability

Data available on request from the authors.

## Supplementary Materials

The following supporting information can be downloaded at https://www.mdpi.com/article/10.3390/cancers18060924/s1. Table S1: Characteristics patients in the cohort 1. Figure S1: Characterization of EVs used in this study. Table S2: List of lectins used in this study. Figure S2: Analyzes of the lectin-positive EVs in NC and PDAC sera with ExoCounter. Figure S3: Comparison of AUC between cohort 1 and cohort 2 for three and four lectins combinations. Figure S4: Precision-Recall-AUC curves, calibration curves, and decision‑curves for data in Figure 4B,C. Figure S5: Box plots of normalized counts for EVs isolated from sera patients of NC (n = 41), PDAC (n = 60), lung (n = 10), colorectal (n = 10), gastric (n = 10), and breast (n = 10) cancers detected with the 11 lectins. Figure S6: Correlation between lectin-positive EVs in cancer sera.

## Abbreviations

The following abbreviations are used in this manuscript:

AAL	Aleuria Aurantia Lectin
ABA	Agaricus bisporus agglutinin
ACA	Amaranthus caudatus agglutinin
ACG	Agrocybe cylindracea galactose-binding lectin
AUC	area under the curve
BC	breast cancer
CC	colorectal cancer
CEA	Carcinoembryonic antigen
ConA	Concanavalin A
DSA	Datura stramonium agglutinin
FBS	fetal bovine serum
FG beads	ferrite-glycidyl methacrylate beads,
GC	gastric cancer
LC	lung cancer
LCA	Lens culinaris agglutinin
LEL	Lycopersicon esculentum lectin
LSL-N	Lathyrus sativus lectin-N
LTL	Lotus tetragon olobus lectin
NC	Normal control
PDAC	Pancreatic ductal adenocarcinoma
PBS	phosphate-buffered solution
PNA	Peanut agglutinin
ROC	receiver operating characteristic
SSA	Sambucus sieboldiana agglutinin
STL	Solanum tuberosum lectin
UDA	Urtica dioica agglutinin
