# Supplementary material for: A Pancreatic Ductal Adenocarcinoma Diagnostic System Using Serum Extracellular Vesicle Detection with Optimized Lectin Combination Using Machine Learning
Source: Cancers (Basel). 2026 Mar 12;18(6):924. doi: 10.3390/cancers18060924 (PMC13024545; doi:10.3390/cancers18060924)
Supplement: Supplementary file 1 [file cancers-18-00924-s001.zip › cancers-4169449-supplementary.pdf]

Table S1. Characteristics patients in the cohort 1.

Parameter	Total (n=42)
**Male gender, n (%)**	29 (69)
**Age, years (median, (range))**	71 (45-85)
**Diabetes mellitus, n (%)**	16 (38)
**Neoadjuvant treatment, n (%)**	23 (55)
**Radiation treatment, n (%)**	7 (17)
**Curative resection, n (%)**	37 (88)
**Pathological stage, n (I /II/III/IV)**	9/26/4/3
**Lymph node metastasis, n (%)**	21 (50)
**Adjuvant chemotherapy, n (%)**	33 (79)
**Preoperative CA19-9>100, n (%)**	15 (41)
**Postoperative CA19-9>100, n (%)**	6 (14)

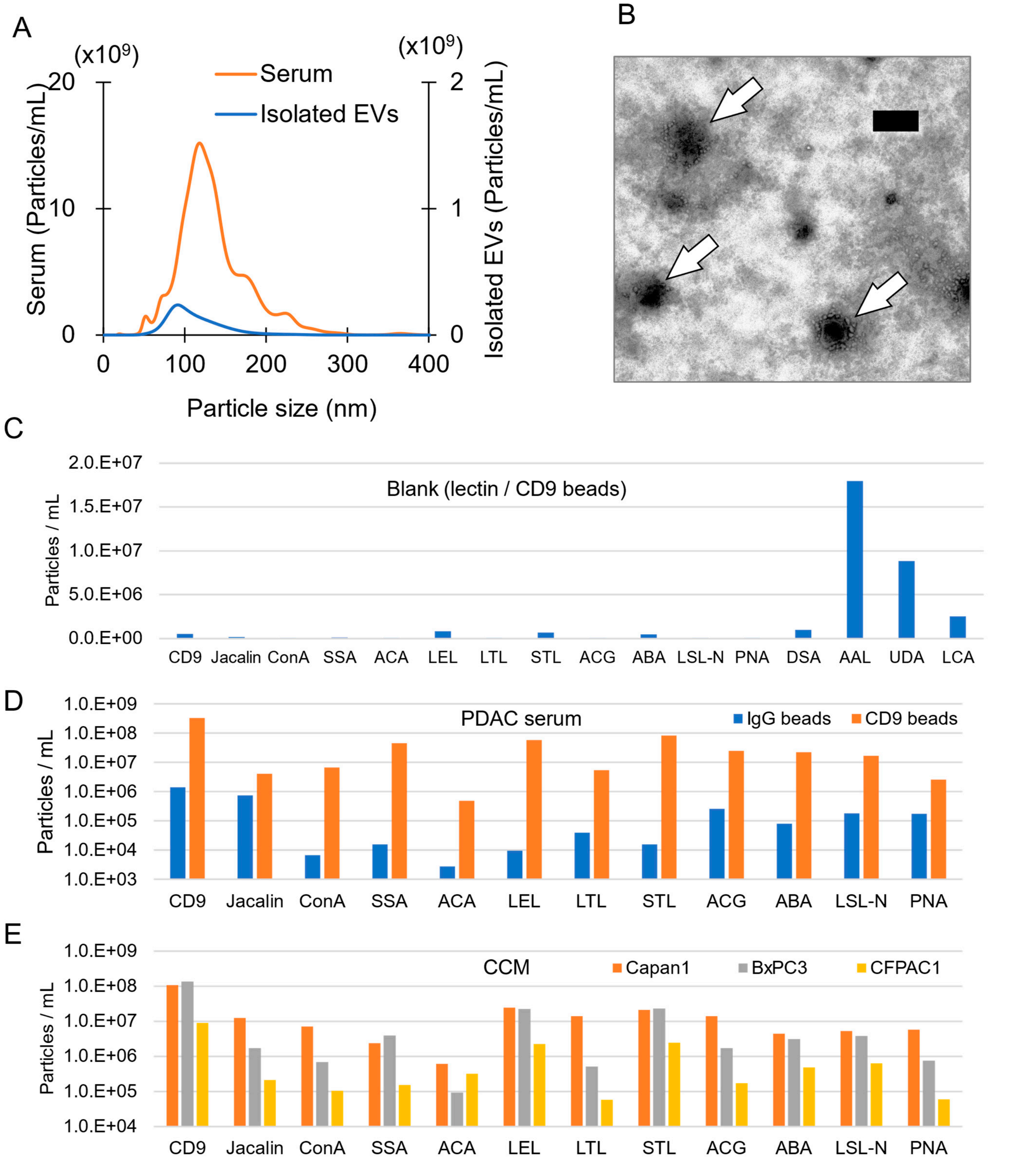

Figure S1. Characterization of EVs used in this study. (A) Size distribution evaluated by NTA for serum and EVs isolated from serum by size exclusion chromatography (SEC). (B) TEM image of the isolated EVs (pointed with white arrows), black bar indicates 100 nm. (C) Adsorption of lectins and anti-CD9. (D) The detection specificity of the lectins with EVs. Discs were coated with the indicated lectins or anti-CD9 antibody, then EV-enriched fractions obtained by SEC from serum of PDAC patient was applied to well followed by labeling with nanobeads conjugated with anti-CD9 or normal IgG antibodies. (E) EVs in the supernatants of cell culture media for Capan1, BxPC3 and CFPAC1 cell lines were detected by using disc coated with anti-CD9 antibody or lectins and nanobeads conjugated with anti-CD9 antibody.

Table S2. List of lectins used in this study.

Name	Specificity	Binding motif	References
Jacalin	Galβ1-3GalNAcα-Thr/Ser (Core1)<br>GlcNAcβ1-3GalNAcα-Thr/Ser (Core3)<br>GalNAcα-Thr/Ser	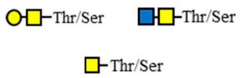	[22–24]
ABA	Galβ1-3GalNAc<br>α 2,3-sialylated Galβ1-3GalNAc/Core2<br>*O*-glycan<br>terminal LacNAc<br>GlcNAcβ-terminated *N*-glycan	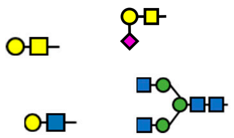	[22,24]
PNA	Galβ1-3GalNAc	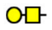	[22–24]
ACA	Galβ1-3GalNAcα-Thr/Ser (Core1)<br>α2,3-sialylated Galβ1-3GalNAcα-Ser/Thr<br>(silyl-Core1)<br>Galβ1-3(GlcNAcβ1-6)α2-6-sialylated<br>GalNAcα-Ser/Thr	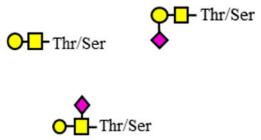	[22,24]
SSA	α2,6-sialylated LacNAc	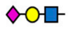	[22,25]
ACG	α2,3-sialylated Lac<br>α2,3-sialylated Galβ1-3GlcNAc<br>α2,3-sialylated Galβ1-3GalNAc<br>polyLacNAc	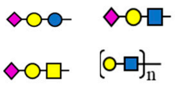	[22,26,27]
ConA	Mannose terminated<br>high-mannose-type *N*-glycan	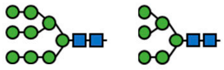	[22,24]
LTL	Fucα1-3GlcNAc, Sia-Lex and Lex	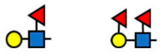	[22,24]
LEL	Type2 polyLacNAc<br>(GlcNAc)n	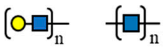	[22,24,28]
STL	Linear internal type2 LacNAc<br>(GlcNAc)n	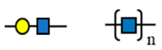	[22,24,28]
LSL-N	polyLacNAc	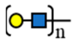	[29]

Sialic acid Glucose Galactose GlcNAc GalNAc Mannose Fucose

SSA, *Sambucus sieboldiana* agglutinin; ACG, *Agrocybe cylindracea* galactose-binding lectin; ACA, *Amaranthus caudatus* agglutinin; PNA, peanut agglutinin; ABA, *Agaricus bisporus* agglutinin; ConA, concanavalin A; LTL, *Lotus tetragonolobus* lectin; LEL, *Lycopersicon esculentum* lectin; STL, *Solanum tuberosum* lectin; LSL-N, *Lathyrus sativus* lectin-N

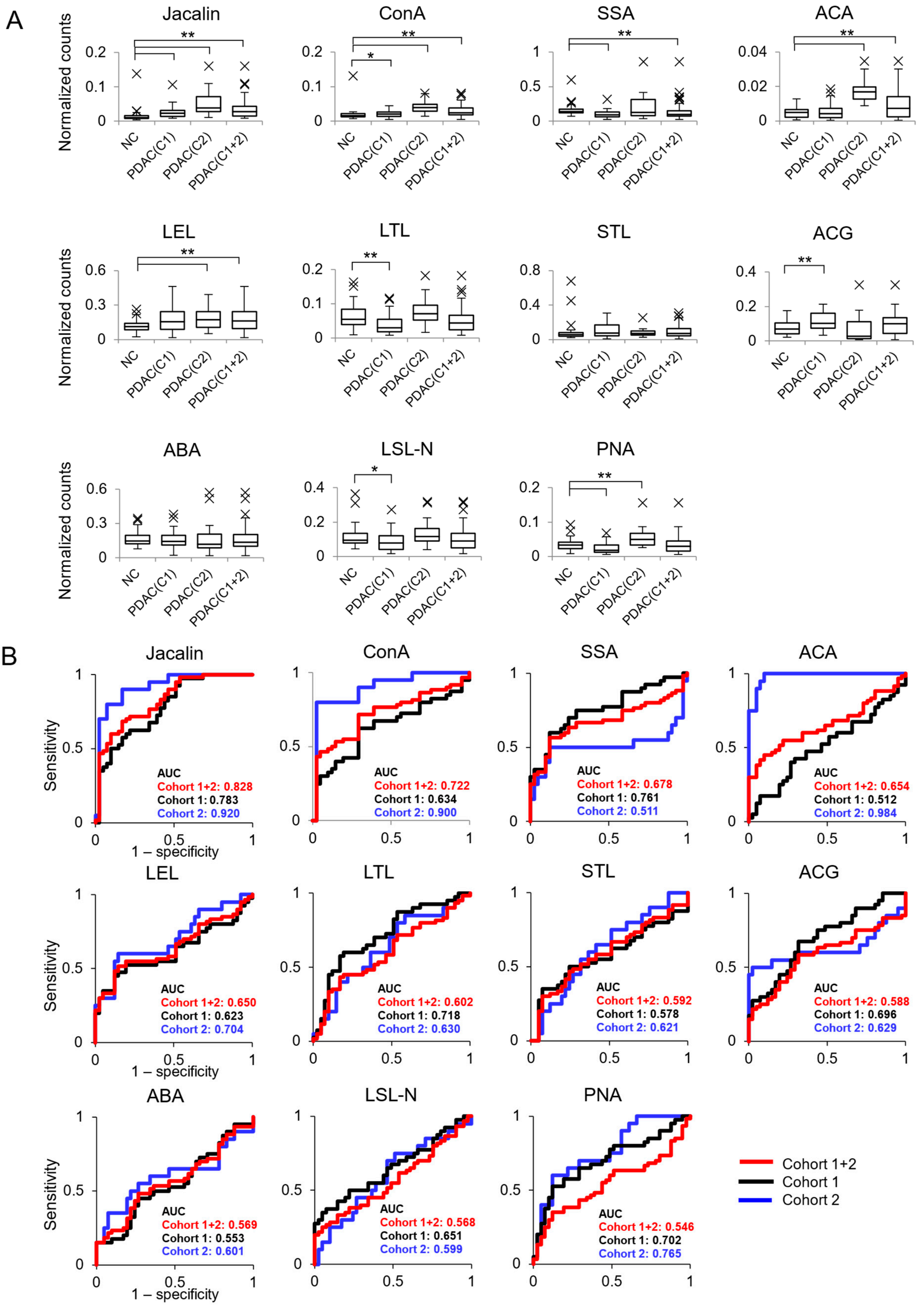

Figure S2. Analyzes of the lectin-positive EVs in NC and PDAC sera with ExoCounter.

(A) The specific EVs in NC n=41, cohort 1 (PDAC n=40), cohort 2 (PDAC n=20) and cohort 1+2 (n=60) were counted using each lectin (Jacalin, ConA, SSA, ACA, LEL, LTL, STL, ACG, ABA, LSL-N, and PNA) with ExoCounter. Each lectin-positive EVs were normalized by the count for the CD9-positive EVs. The P-values were calculated by ANOVA (*P<0.05 and **P<0.01). (B) ROC curves for lectin-positive EVs from PDAC compared to those from NC.

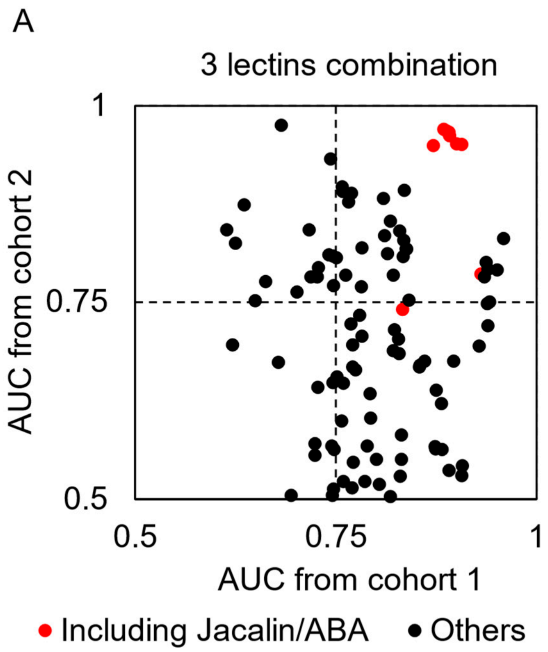

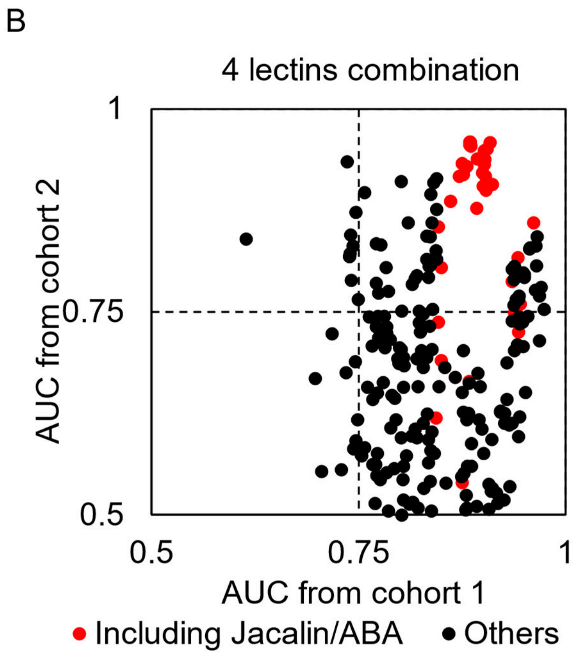

Lectin			Cohort 1	Cohort 2	Cohort 1+2
Jacalin	ABA	PNA	0.906	0.951	0.921
Jacalin	ABA	LTL	0.900	0.952	0.917
Jacalin	LEL	SSA	0.959	0.832	0.916
Jacalin	ABA	LSL-N	0.890	0.967	0.916
Jacalin	ABA	ACA	0.891	0.962	0.915
Jacalin	ABA	LEL	0.887	0.968	0.914
Jacalin	ABA	STL	0.884	0.971	0.913
Jacalin	STL	SSA	0.951	0.791	0.898
Jacalin	ABA	ConA	0.871	0.950	0.897
Jacalin	ConA	SSA	0.937	0.801	0.891

Lectin				Cohort 1	Cohort 2	Cohort 1+2
Jacalin	ABA	SSA	LEL	0.961	0.860	0.946
Jacalin	ABA	SSA	STL	0.950	0.796	0.944
Jacalin	ABA	SSA	ConA	0.938	0.806	0.941
Jacalin	ABA	SSA	LSL-N	0.935	0.788	0.939
Jacalin	ABA	SSA	LTL	0.945	0.760	0.937
Jacalin	ConA	SSA	STL	0.949	0.793	0.925
Jacalin	ACG	SSA	LEL	0.957	0.829	0.922
Jacalin	ABA	STL	ConA	0.871	0.917	0.922
Jacalin	ConA	SSA	LEL	0.956	0.828	0.921
Jacalin	ConA	SSA	LSL-N	0.935	0.802	0.920

Figure S3. Comparison of AUC between cohort 1 and cohort 2 for three (A) and four (B) lectins combinations. The tables represent the best (top 10) AUC results from cohorts 1+2. The combinations containing Jacalin/ABA are indicated by red dots.

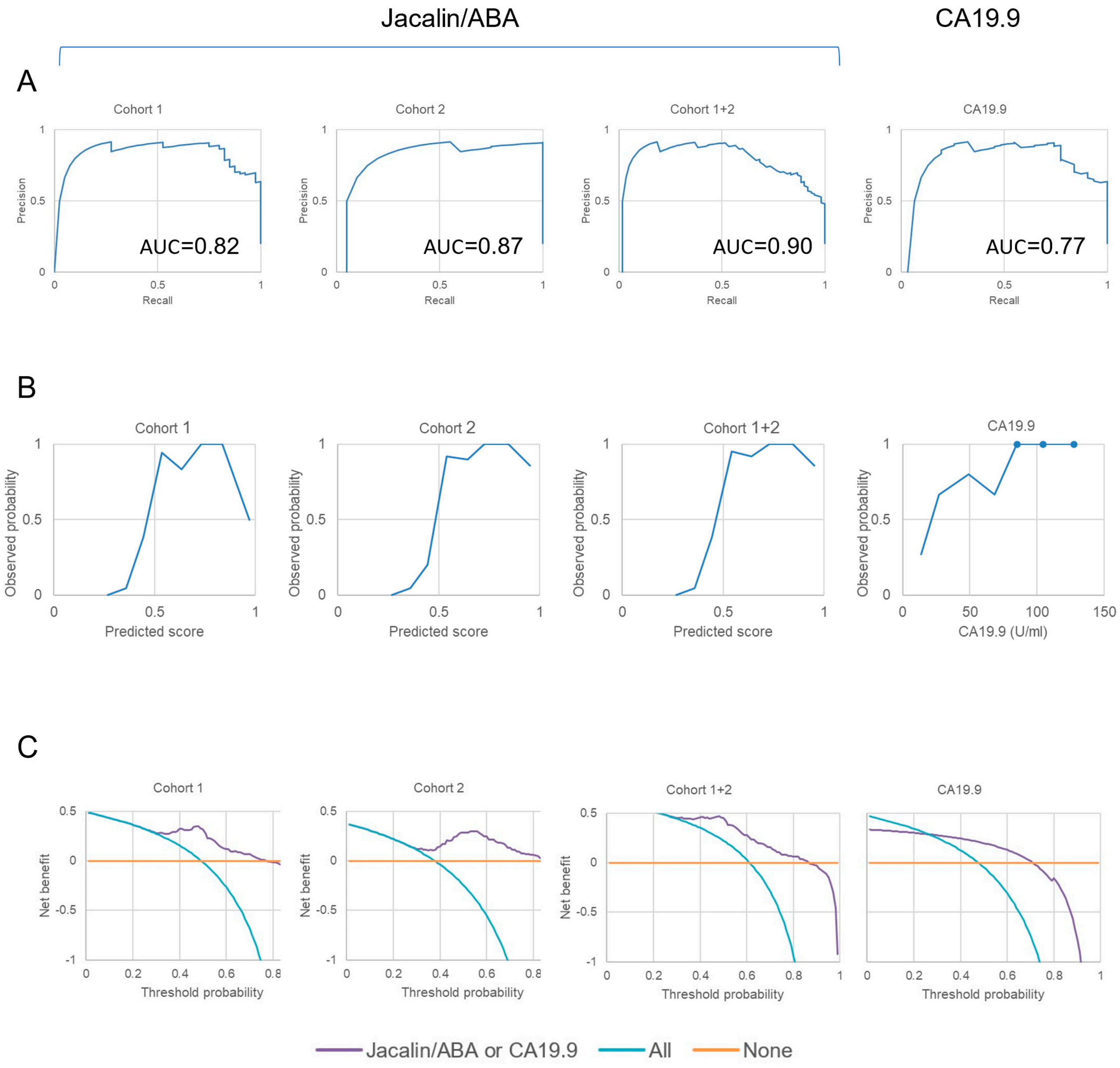

Figure S4. Precision-Recall-AUC curves (A), calibration curves (B), and decision-curves (C) are analyzed from the data in Figure 4B and C.

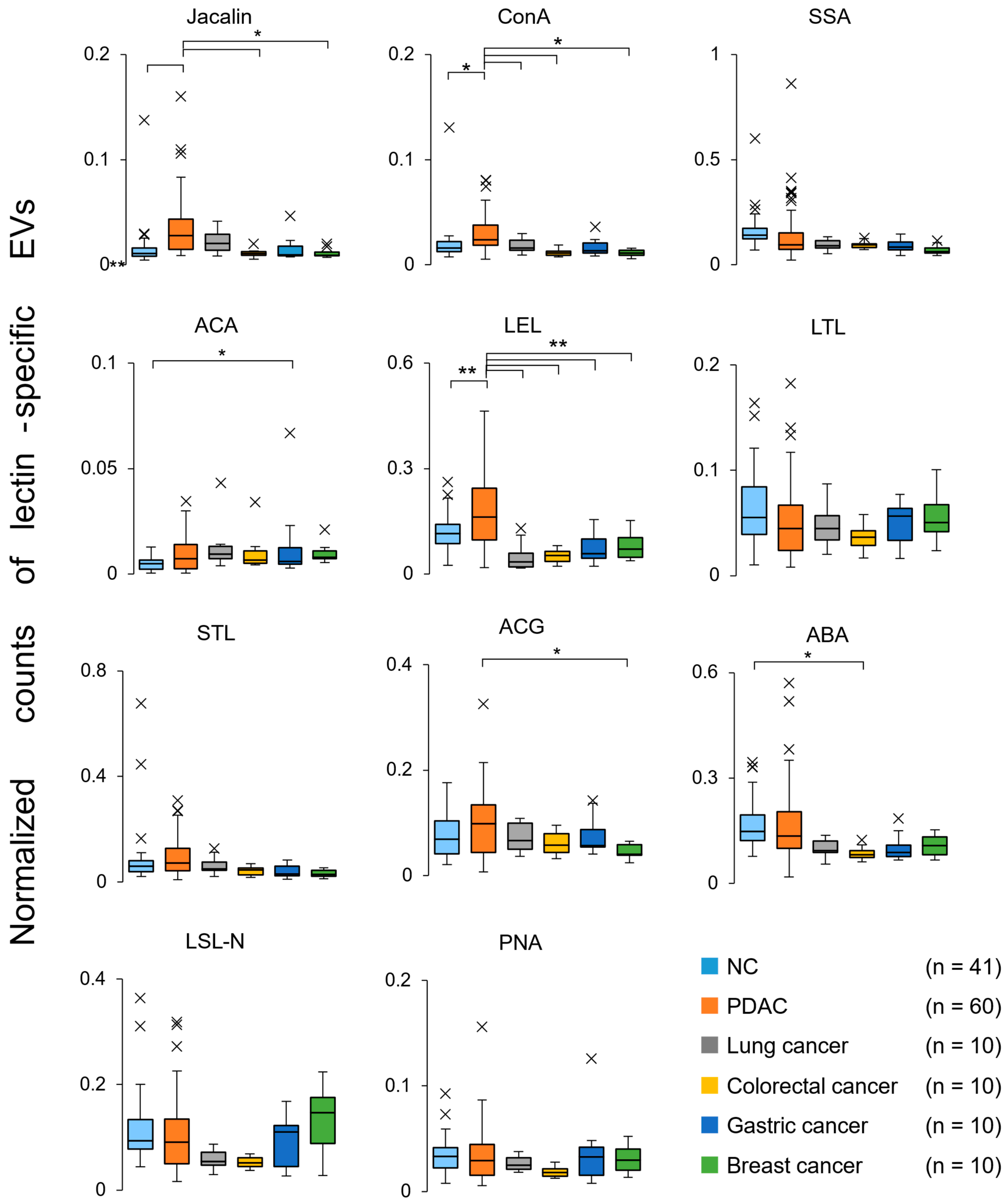

Figure S5. Box plots of normalized counts for EVs isolated from sera patients of NC (n=41), PDAC (n=60), lung (n=10), colorectal (n=10), gastric (n=10), and breast (n=10) cancers detected with the 11 lectins. Statistical analysis was performed using ANOVA with the Tukey-Kramer test (*P<0.05, **P<0.01).

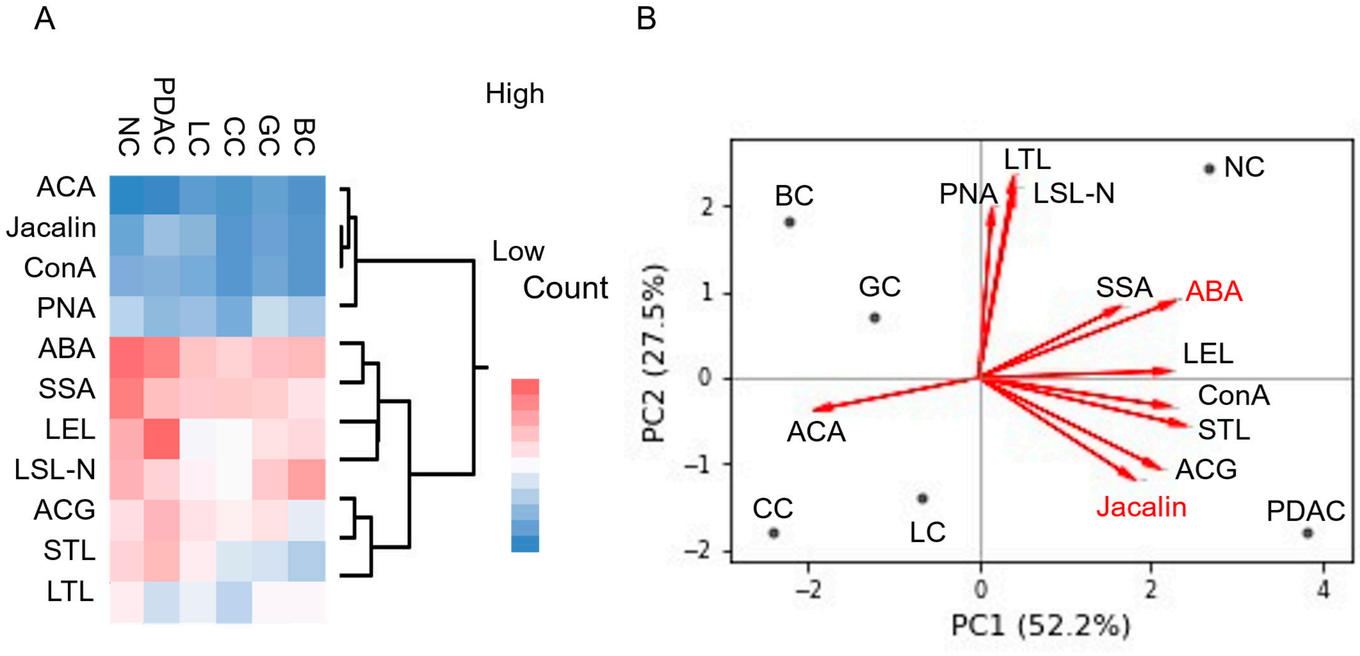

Figure S6. Correlation between lectin-positive EVs in cancer sera. (A) Multivariable analysis was performed using the data of PDAC, lung, colorectal, gastric, or breast cancers and NC for each lectin. The graph shows the data from heat map analysis and hierarchical clustering analysis. (B) The biplot shows the results of the principal component analysis.
